# Low SARS-CoV-2 seroprevalence in blood donors in the early COVID-19 epidemic in the Netherlands

**DOI:** 10.1038/s41467-020-19481-7

**Published:** 2020-11-12

**Authors:** Ed Slot, Boris M. Hogema, Chantal B. E. M. Reusken, Johan H. Reimerink, Michel Molier, Jan H. M. Karregat, Johan IJlst, Věra M. J. Novotný, René A. W. van Lier, Hans L. Zaaijer

**Affiliations:** 1 Department of Blood-borne Infections, Sanquin Research, Amsterdam, The Netherlands; 2 Department of Medical Affairs, Sanquin Corporate Staff, Amsterdam, The Netherlands; 3 Department of Virology, Sanquin Diagnostic Services, Amsterdam, The Netherlands; 4 Centre for Infectious Disease Control, National Institute for Public Health and the Environment, Bilthoven, The Netherlands; 5 Department of Viroscience, Erasmus MC, Rotterdam, The Netherlands; 6 Department of Donor Studies, Sanquin Research, Amsterdam, The Netherlands; 7 National Screening Laboratory, Sanquin Laboratory Services, Amsterdam, The Netherlands; 8 Department of Medical Affairs, Sanquin Blood Bank, Amsterdam, The Netherlands; 9 Landsteiner Laboratory, Sanquin Research, Amsterdam, The Netherlands; 10 Department of Experimental Immunology, University of Amsterdam, Amsterdam, The Netherlands; 11 Department of Medical Microbiology, Amsterdam UMC, Amsterdam, The Netherlands

**Keywords:** Viral infection, Infectious-disease diagnostics, SARS-CoV-2, Epidemiology

## Abstract

The world is combating an ongoing COVID-19 pandemic with health-care systems, society and economies impacted in an unprecedented way. It is unclear how many people have contracted the causative coronavirus (SARS-CoV-2) unknowingly and are asymptomatic. Therefore, reported COVID-19 cases do not reflect the true scale of outbreak. Here we present the prevalence and distribution of antibodies to SARS-CoV-2 in a healthy adult population of the Netherlands, which is a highly affected country, using a high-performance immunoassay. Our results indicate that one month into the outbreak (i) the seroprevalence in the Netherlands was 2.7% with substantial regional variation, (ii) the hardest-hit areas showed a seroprevalence of up to 9.5%, (iii) the seroprevalence was sex-independent throughout age groups (18–72 years), and (iv) antibodies were significantly more often present in younger people (18–30 years). Our study provides vital information on the extent of exposure to SARS-CoV-2 in a country where social distancing is in place.

## Abstract (web summary)

The Netherlands is a country highly affected by the COVID-19 pandemic. In this study, Slot, Hogema and colleagues report a low SARS-CoV-2 seroprevalence one month into the outbreak and provide insights into virus exposure by region and age group when widespread non-pharmaceutical interventions are in place.

## Introduction

Coronavirus disease 2019 (COVID-19) is an emerging infectious disease caused by the severe acute respiratory syndrome coronavirus 2 (SARS-CoV-2). The disease was first documented in humans in China in December 2019. The virus has since spread globally by person-to-person transmission, resulting in an ongoing pandemic impacting public health, health-care systems, society and the economy across the world^1–4^.

Common clinical COVID-19 manifestations include fever, cough, fatigue, expectoration, shortness of breath, dyspnoea and muscle soreness^5^. The median incubation period is estimated to be 5.1 days (95% CI, 4.5 to 5.8 days), and 97.5% of those who develop symptoms will do so within 11.5 days (95% CI, 8.2 to 15.6 days) of infection^6^. While the majority of patients show only mild or moderate symptoms, some progress to viral pneumonia, acute respiratory distress syndrome (ARDS), systemic inflammatory response syndrome (SIRS), multiple organ failure (MOF) and death.

As of 24 June 2020 more than 9.1 million laboratory-confirmed COVID-19 cases have been reported, including 473,797 deaths, affecting 215 countries and territories around the world^4^. The reported COVID-19 cases do not reflect the true scale of outbreak^7–11^. In many countries lockdown strategies have been implemented and social distancing is mandatory to reduce person-to-person transmission, protecting citizens and mitigating the impact on health-care systems including intensive care capacities. Likely, social distancing measures will need to remain in place until population-based immunity is achieved through natural exposure to the virus (herd immunity) or until effective vaccines or therapeutics become available^12,13^.

If immune responses to SARS-CoV-2 are similar when compared to other coronaviruses, infected individuals may be less susceptible to reinfection for months to years, reducing the risk of severe COVID-19 and also limiting the possibility of spreading the virus^14,15^. Infection-induced herd immunity might arise when enough people become infected and develop antibodies to the virus, in addition to people with non-antibody-mediated immunity or with SARS-CoV-2 pre-existing immune reactivity^16,17^. Above the herd immunity threshold (HIT), which represents a minimum number of people in a community that would need to be immune, the infection may no longer persist in the population. According to basic models, the HIT of SARS-CoV-2 is estimated at 50 to 67%^18,19^. This relies on simplified assumptions, such as homogeneous population mixing and uniform sterilizing immunity in recovered individuals across demographic groups, which are unlikely to hold true. Nevertheless, the HIT provides an indication of the minimum proportion of a population that would need to be immune until herd immunity could be achieved in the absence of a vaccine.

It is unclear how many people contract the virus unknowingly. Assuming that antibodies to SARS-CoV-2 are produced by the adaptive immune system in response to virus exposure, at least in the vast majority of cases, serology-based tests for SARS-CoV-2 may be used to determine the extent of asymptomatic SARS-CoV-2 infections and to monitor the COVID-19 pandemic. Highly accurate immunoassays were not available until recently^15,20,21^.

Population-based serological studies as well as high-quality data on SARS-CoV-2 antibody production in healthy individuals are urgently needed to assess both the true extent of virus spread and the presence of potential antibody-mediated protection against SARS-CoV-2 at the community level. Using outbreak and pre-outbreak samples, we studied the prevalence and distribution of antibodies to SARS-CoV-2 under social distancing in a healthy adult population of a highly affected country, one month into the outbreak.

Here, we show a low SARS-CoV-2 seroprevalence in the early COVID-19 epidemic in the Netherlands, demonstrating that (i) the seroprevalence was 2.7% with substantial regional variation, (ii) the hardest-hit areas showed a seroprevalence of up to 9.5%, (iii) the seroprevalence was sex-independent throughout age groups (18–72 years), and (iv) antibodies were significantly more often present in younger people (18–30 years).

## Results

### Serological test results

In total 7,361 donations from regular plasma donors were tested, of which 248 (3.4%) were initially reactive and 230 (3.1%) were repeat reactive in the Wantai total antibody assay. For 218/230 repeat reactive donors archived material of a pre-outbreak donation was available for testing, showing seroconversion in 188/218 donors (86%) and pre-outbreak reactivity in 30/218 (14%); for 12 repeat reactive donors no pre-outbreak samples were available. The 188 donors with confirmed seroconversion and the 12 donors from whom no pre-outbreak sample was available were considered seropositive in the subsequent analyses. Positive IgM test results were found as follows: in 144/180 (80%) seroconverters (8 donors not tested); in 3/28 (11%) donors with pre-outbreak reactivity (2 donors not tested); and in 8/12 (67%) donors from whom no pre-outbreak sample was available.

### Distribution of SARS-CoV-2 antibody signals

**Fig. 1:**
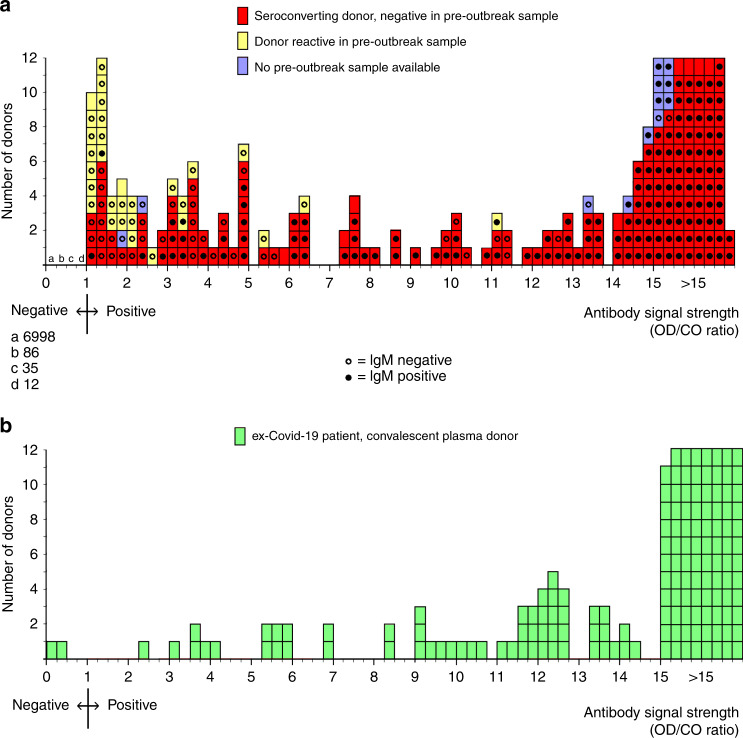
Distribution of SARS-CoV-2 antibody signals in regular blood plasma donors and in recovered COVID-19 patients donating convalescent plasma; 1–15 April 2020. **a** Distribution of SARS-CoV-2 total antibody signals and SARS-CoV-2 IgM test results in regular blood plasma donors (*n* = 7,361). **b** Distribution of SARS-CoV-2 total antibody signals in recovered COVID-19 patients donating convalescent plasma (*n* = 153).

The distribution of SARS-CoV-2 total antibody signals in positive donors (*n* = 230) and negative donors (*n* = 7,131) is shown in Fig. 1a. Details about seroconversions and false-reactive test results can be found in Supplementary Fig. 1. As a reference, the SARS-CoV-2 total antibody signals in ex-COVID-19 patients donating convalescent plasma (*n* = 153) are shown in Fig. 1b. These patients had a documented PCR-positive test result, were at least 14 days fully recovered from mild to severe COVID-19 symptoms and were sampled in the study period. The distribution of SARS-CoV-2 total antibody signals in ex-COVID-19 patients was comparable, but not identical, to regular plasma donors who seroconverted; low-positive signals were found more often in regular plasma donors than in ex-COVID-19 patients.

### Seroprevalence by geographic region, sex and age group

**Fig. 2:**
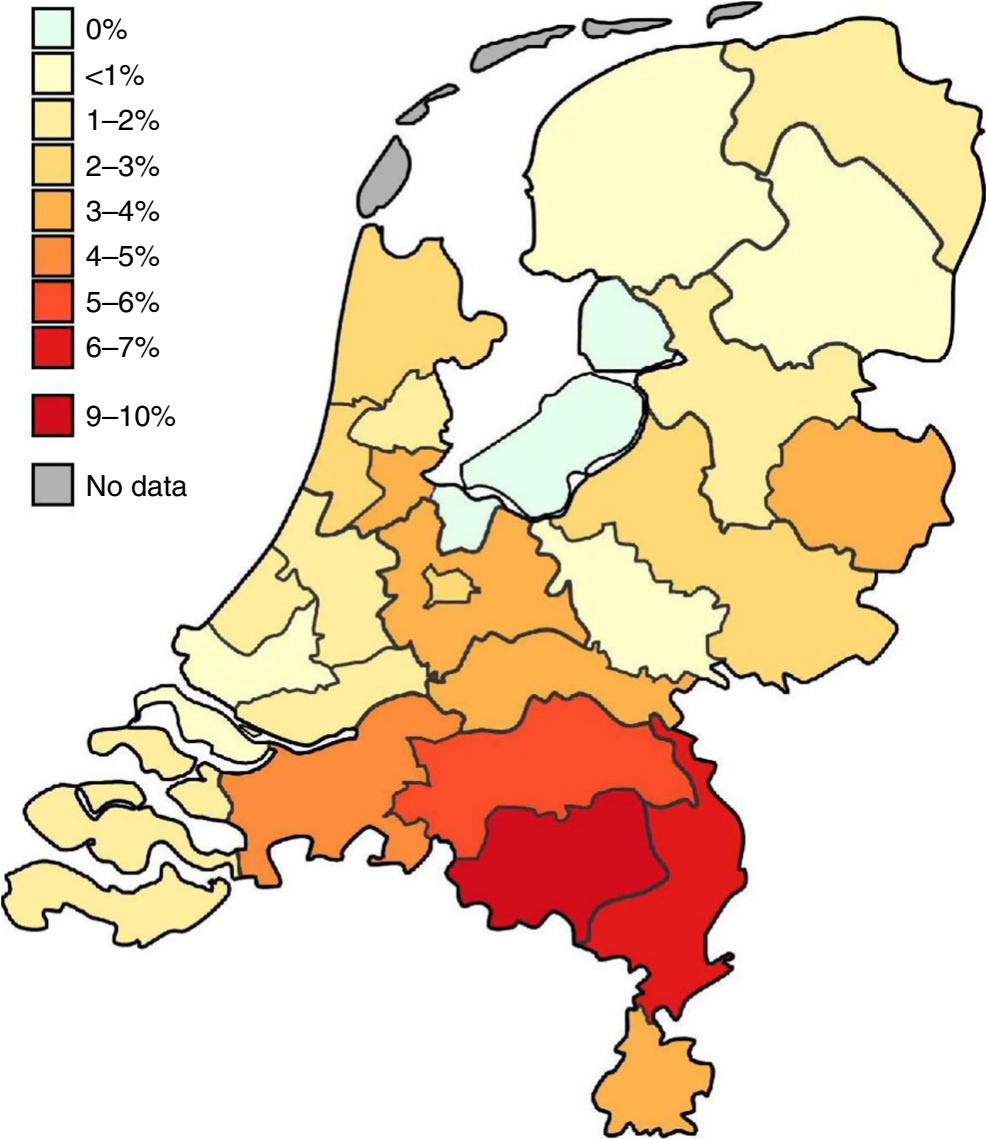
SARS-CoV-2 seroprevalence in the Dutch blood donor population by municipal health service region; 1–15 April 2020. The map shows the Netherlands divided into municipal health service regions. The backbone of the map was provided by the Dutch Institute for Public Health and the Environment.

Based on demonstrated seroconversion, 188/7,361 (2.6%) donors have been infected with SARS-CoV-2. If donors from whom no pre-outbreak sample was available are included this number is 200/7,361 (2.7%). The seroprevalence varied by geographic region. Figure 2 shows the seroprevalence in the 26 municipal health service regions of the Netherlands, demonstrating a north to south gradient. Details about the seroprevalence by region, sex and age group can be found in Supplementary Table 1. The prevalence of antibodies to SARS-CoV-2 was not different for men and women (2.70% vs 2.73%). Logistic regression and chi-square analysis showed that the seroprevalence in donors aged 18–30 years was significantly higher than in other age groups (4.2% vs 2.4%; *p* = 0.026; Table 1). No additional association with sex or age group was found in the logistic regression model if the data were corrected for region of residence.

**Table 1:** Prevalence of anti-SARS-CoV-2 antibodies in Dutch blood plasma donors (*n* = 7,361) by age group; 1–15 April 2020.

Age group (years)	Anti-SARS-CoV-2 prevalence	95% confidence interval
18–30	52/1,251 (4.2%)	3.1–5.4%
31–40	22/882 (2.5%)	1.6–3.8%
41–50	31/1,354 (2.3%)	1.6–3.2%
51–60	48/2,132 (2.3%)	1.7–3.0%
61–72	47/1,742 (2.7%)	2.0–3.8%

## Discussion

The world is facing unprecedented challenges with communities and economies everywhere affected by the COVID-19 pandemic. Social distancing and other public health measures are placing an increasing psychological burden on people, resulting in an increasing need for exit strategies^22^.

As the situation evolves, public health interventions significantly lower the real-time reproduction number (*R*_t_), which estimates the average number of secondary cases one case produces in a population made up of both susceptible and non-susceptible hosts^23^. If person-to-person transmission is limited and the *R*_t_ drops, the pandemic may be controlled on a population level until an effective SARS-CoV-2 vaccine or pharmacological therapy for COVID-19 becomes available^12,13^.

Another way out may be acquiring natural herd immunity to SARS-CoV-2 as an indirect protection conferred by immune individuals to the susceptible ones in the population^18,19,24^. If natural herd immunity to SARS-CoV-2 is attainable during the pandemic, policy makers may take this into account as an exit strategy option. The 2.7% seroprevalence found in our study shows that, 1 month into the outbreak and more than 2 weeks after social distancing and lockdown interventions were implemented, the proportion of SARS-CoV-2 antibody-positive individuals in the population tested was far below the 50–67% HIT. Without social distancing the chains of infection may resume shortly, even in the hardest-hit areas with a seroprevalence of up to 9.5%. Importantly, recently published population-based seroprevalence studies in areas of other countries, using different immunoassays and divergent methods of sampling, also show low prevalences of antibodies to SARS-CoV-2, suggesting that populations are likely to remain susceptible to the virus and future waves of the outbreak are inevitable without a vaccine or antiviral prophylaxis^25–27^.

Antibodies were significantly more often detected in younger people (18–30 years), which might be related to age-dependent social behaviours before social distancing was implemented. Our study further indicates that the prevalence of antibodies to SARS-CoV-2 is sex-independent throughout age groups (18–72 years) and can vary substantially among areas in a country. The regional variation in the Netherlands may be associated with the celebration of Carnival, for which main events occurred in the south and southeastern parts of the country during the last week of February 2020, when social distancing and restrictions on public events and gatherings were not in place yet. Of course, the study only covers SARS-CoV-2 infections in the first month of the outbreak, and the results cannot be extrapolated to children, non-healthy adults and elderly aged >72 years.

Our study was performed in the early COVID-19 epidemic in the Netherlands. The 2.7% seroprevalence in the population tested demonstrates limited presence of potential antibody-mediated immunity to SARS-CoV-2 at the time of the study. Because antibodies to the virus may not be detected during the first weeks post infection, it can be assumed that more people were infected between 27 February 2020 (first Dutch COVID-19 case) and April 2020 (the moment of sampling). Therefore, the presence of potential antibody-mediated immunity may have been higher than 2.7%. Using the same assay, the SARS-CoV-2 seroprevalence in the Dutch donor population had increased to 5.9% (419/7,150) in May 2020^28^. This indicates that the proportion of SARS-CoV-2 antibody-positive individuals in the Netherlands remained far below the 50–67% HIT during the first months of the COVID-19 epidemic. Of course, acquiring herd immunity may also depend on both SARS-CoV-2 pre-existing immune reactivity and vaccine-induced herd immunity, when a COVID-19 vaccine becomes available^29^.

To check for potential selection bias due to using regular plasma donors as study subjects, we calculated the prevalence of antibodies to anti-SARS-CoV-2 after correction for the number of residents in the various municipal health regions and for the age distribution of the Dutch general population (18–70 years). Although the number of donations tested varied between the municipal health regions from 0.83 to 8.27 per 10,000 inhabitants, the weighted seroprevalence was 2.73%, which is identical to the unadjusted seroprevalence. Similarly, the estimated weighted seroprevalence of the Dutch general population (20–70 years) was 2.76%, indicating that differences in the age distribution of the plasma donor population studied, compared with the Dutch general population, had no significant impact on the study results. Further, the observed regional variation in SARS-CoV-2 seroprevalence matches well with the reported geographic variation of both laboratory-confirmed and hospitalized COVID-19 cases in the Netherlands^30^. We therefore consider the seroprevalence found in a sample of the Dutch donor population as an important indication of the seroprevalence in the general population of the Netherlands, until more specific data becomes available.

The Wantai SARS-CoV-2 total antibody ELISA shows performance characteristics superior to other immunoassays currently on the market^31,32^. We calculated a test specificity of 99.6% using 282 pre-outbreak samples collected in March and April 2018 (validation data in Method section). In the seroprevalence study we found false-reactive test results in 30/7361 (0.4%) subjects. For these subjects, false reactivity was present before the COVID-19 outbreak with stable OD/CO ratios over months (Supplementary Fig. 1), indicating consistent non-specific reactions or cross-reactivity with antibodies to common seasonal coronaviruses (OC43, 229E, NL63, HKU1)^33^. It is unknown whether this potential cross-reactivity provides cross-protection against SARS-CoV-2 infection.

Of note, the positive predictive value (PPV) of a test depends on the combination of test specificity and seroprevalence. In our study, the PPV of the Wantai SARS-CoV-2 total antibody ELISA was 99%, 88% and 72% in areas with a seroprevalence of 4–10%, 2–4% and <2%, respectively. Hence, the PPV increases with the seroprevalence and will increase as the outbreak proceeds. This phenomenon must be taken into account if SARS-CoV-2 antibody testing would in the future be considered for diagnostic services and for reducing social distancing measures.

Our study has several limitations. First, samples were collected from regular plasma donors without further selection. Although all subjects were healthy at the time of sampling and did not report health issues in the 2 weeks before, they might have been recovered from COVID-19 symptoms earlier. As a consequence, the number of subjects enrolled in the study after full recovery from COVID-19 symptoms is unknown. It is therefore not possible to specifically assign SARS-CoV-2 seroprevalence data and total antibody signals to asymptomatic or symptomatic infections. Second, donors who experience COVID-19 symptoms may not recover within a few weeks, which might have resulted in self-deferral and not showing up for plasma donation. Therefore, donors who suffered from COVID-19 symptoms may be underrepresented in the study. Third, donors who experienced COVID-19 symptoms during the 2 weeks before sample collection were not eligible to donate plasma in accordance with European laws and guidelines^34,35^. They were not enrolled in the study, resulting in a possible underestimation of the seroprevalence in the donor population. Fourth, the test sensitivity in PCR-confirmed COVID-19 patients, who were at least 14 days fully recovered when sampled, was 98.7% (151/153 seropositive; Fig. 1b). This demonstrates that antibodies to SARS-CoV-2 were detected in nearly all COVID-19 patients after full recovery from mild to severe symptoms. However, the test sensitivity in asymptomatic cases is unknown and might be lower than in symptomatic cases, which could have resulted in an underestimation of the seroprevalence in the donor population.

It is unclear whether the presence of SARS-CoV-2 antibodies in blood reflects immunity, be it short term or long term^15,33,36^. Recent studies found strong correlation between ELISA results and virus neutralization^37,38^. Wu and colleagues studied the levels and time course of neutralizing antibodies (nAbs) in 175 patients who experienced mild COVID-19 symptoms^39^. In those patients nAbs were detected from days 10–15 after onset of disease and remained thereafter. Interestingly, middle-aged and elderly patients had significantly higher nAb titres (*p* < 0.0001) than younger patients. Ten patients had undetectable nAb titres (ID50: <40) and two showed very high titres (ID50: 15,989 and 21,567). These findings indicate that antibodies to SARS-CoV-2 in asymptomatic individuals and in patients with only mild symptoms may have limited neutralizing capacity. Like in other infections, non-nAbs may play a significant role in decreasing the viral load, e.g. via Fc receptor-mediated uptake in innate cells, leading to partial or even total protection from reinfection^40^. Therefore, SARS-CoV-2 antibody-mediated immunity, including the role and formation of nAbs and non-nAbs, needs further research both to ascertain whether asymptomatic and non-severe cases become immune to the virus and to assess the relevance of SARS-CoV-2-specific antibodies for the development of safe and effective (i) vaccines, (ii) treatment of COVID-19 patients with convalescent plasma transfusions, and (iii) plasma-derived medicinal products (anti-SARS-CoV-2 hyperimmune globulins)^12,13,19,41–43^.

Gilbert and colleagues have proposed an exit strategy approach in which at first only seropositive individuals, recovered from SARS-CoV-2 infection, return to their normal lives^44^. When the pandemic subsides, gradually younger low-risk people without symptoms might follow. Such an approach would slowly build up immunity in the population, mitigating the impact on health-care systems and intensive care capacities and reducing the intensity of future waves of the pandemic. This would make it possible to reconcile the advantages of two opposing strategies that have been proposed: the strategy of global containment of the population, which is economically and socially costly, and the strategy based on natural herd immunity, which potentially involves a substantial human cost if done too fast. To put this approach in context, we stress that the protective effect of SARS-CoV-2-specific antibodies in blood is not yet known and that serology-based tests in general come with important pitfalls until accurate confirmatory testing is available^15,33,36,45^. An antibody testing-based exit strategy could thus lead to a resurgence of community spread of the virus.

In conclusion, the seroprevalence in the healthy adult population shows no evidence of adequate antibody-mediated immunity to SARS-CoV-2 on the country level, suggesting that acquiring natural herd immunity is not a realistic COVID-19 exit strategy in the short term. Downscaling public health interventions, including social distancing, in the absence of population-based immunity may significantly increase the *R*_t_ and fuel the COVID-19 pandemic, possibly resulting in uncontrollable virus spread. During the pandemic it is pivotal to continuously revisit public health interventions and lockdown exit strategies using up-to-date data on both community spread of SARS-CoV-2 and immunity to the virus.

## Methods

### Setting

The Netherlands is the most densely populated country of Europe with 17.2 million human inhabitants (421 per square kilometre). The first Dutch case of COVID-19 was reported on 27 February 2020. An outbreak ensued, culminating in 812, 5159, 9127 and 11,863 hospital admissions, and in 1868, 13,614, 28,153 and 49,914 PCR-confirmed COVID-19 cases including 64, 1173, 3134 and 6100 documented fatalities by 15 March, 1 April, 15 April and 24 June 2020, respectively^46^. Social distancing and lockdown interventions were implemented nationwide on 15 March 2020, when all restaurants, cafés, bars, discotheques, coffeeshops, musea, concert halls, theatres, sports clubs, saunas, sex clubs, universities, schools and childcare centres were closed and specific restrictions on public events, gatherings, work and travel became mandatory^47^. The Dutch approach was comparable to most other European countries and all public health measures were well respected.

### Subjects and sampling

We studied the plasma samples of 7361 regular blood plasma donors from throughout the Netherlands, collected from 1 until 15 April 2020. Subjects were enrolled in the study if they were accepted for routine donation, without further selection and in accordance with European laws and guidelines^34,35^. As a consequence, all subjects were healthy at the time of donation and had not reported health issues in the 2 weeks before donation. Subjects were defined by age (18–72 years), gender and zip code of the subject’s residence. All age groups were well represented with a balanced distribution of male and female subjects residing in all regions of the Netherlands.

### Serological testing

Donor samples were screened for antibodies to SARS-CoV-2 using a SARS-CoV-2 total antibody ELISA (Wantai Biological Pharmacy Enterprise Co., Ltd., Beijing, China) following the manufacturer’s instructions. Samples testing reactive (OD/CO ratio ≥ 1) were re-tested and considered positive if the re-test was reactive. For the majority of positive donors an archived sample of a donation from before the start of the COVID-19 outbreak was available and tested, to determine seroconversion or false reactivity. For this purpose, seroconversion was defined as transition from negative to positive with at least a two-fold increase in OD/CO ratio. To study the consistency of false-reactive test results, archived samples of multiple associated pre-outbreak donations were tested, if available. Briefly, the Wantai ELISA used is a ‘double antigen sandwich assay’. This assay format has the following advantages: the solid phase is coated with recombinant SARS-CoV-2 antigens, which simultaneously bind antibody isotypes (IgA, IgM, IgG) directed to SARS-CoV-2. For detection, a labelled SARS-CoV-2 antigen is used. Additional testing of samples that were positive in the SARS-CoV-2 total antibody ELISA was performed using a SARS-CoV-2 IgM ELISA (Wantai Biological Pharmacy Enterprise Co., Ltd., Beijing, China) following the manufacturer’s instructions.

### Validation and evaluation

Performance characteristics of the Wantai SARS-CoV-2 total antibody ELISA have been assessed in third-party evaluations, indicating a sensitivity of 100% in PCR-confirmed COVID-19 cases (after 15–39 days following the onset of symptoms; *n* = 90) and a specificity of 99.1% (*n* = 213) to 100% (*n* = 82)^31,32^. The performance of the Wantai ELISA, including positive and negative predictive values, was superior compared to other assays^32^. We additionally validated the Wantai ELISA using panels of plasma and serum samples from (i) Dutch blood donors collected in March and April 2018 (*n* = 282; 1/282 seropositive), (ii) PCR-confirmed COVID-19 patients admitted to Dutch intensive care units in March 2020 (*n* = 10; 9/10 seropositive; 1/10 seronegative), (iii) Dutch PCR-confirmed COVID-19 patients with only mild symptoms (*n* = 11; 11/11 seropositive), (iv) Dutch donors with a documented PCR-positive test result donating convalescent plasma, who were at least 14 days fully recovered from mild to severe COVID-19 symptoms (*n* = 153; 151/153 seropositive), and (v) patients with PCR-positive test results for common HCoV, CMV or EBV infection (*n* = 40; 0/40 seropositive).

### Data collection and analysis

Test results were obtained as described above. The age, gender and zip code of residence of the individual subjects (both regular plasma donors and recovered COVID-19 patients donating convalescent plasma) were retrieved from the blood bank information system ePROGESA v5.0.3 (MAK-SYSTEM International Group, France). Data were processed and analyzed using Microsoft Office Access version 16.0 (Microsoft Corporation, USA). Association between the prevalence of anti-SARS-CoV-2 antibodies and age, sex and region of residence was assessed by logistic regression analysis using R v.3.5.2 (R Foundation, Austria).

### Ethics statement

Samples were collected only from voluntary, non-remunerated, adult donors who provided written informed consent as part of routine donor selection and blood collection procedures. The study was reviewed and approved by the Ethics Advisory Council of Sanquin Blood Supply Foundation on March 27, 2020.

### Reporting Summary

Further information on research design is available in the Nature Research Reporting Summary linked to this article.

## Supplementary information

Supplementary Information

Reporting Summary

## Data Availability

The authors declare that all data supporting the findings of this study are available within the paper and its supplementary information files. More detailed information on research data is available from the corresponding authors upon reasonable request. Source data are provided with this paper.

## Source data

Source Data
