# Supplementary material for: Low SARS-CoV-2 seroprevalence in blood donors in the early COVID-19 epidemic in the Netherlands
Source: Nat Commun. 2020 Nov 12;11:5744. doi: 10.1038/s41467-020-19481-7 (PMC7665189; doi:10.1038/s41467-020-19481-7)
Supplement: Supplementary file 1 — Supplementary Information [file 41467_2020_19481_MOESM1_ESM.pdf]

SUPPLEMENTARY INFORMATION

Low SARS-CoV-2 seroprevalence in blood donors in the early COVID-19 epidemic in the Netherlands

Ed Slot, Boris M. Hogema, Chantal B.E.M. Reusken, Johan H. Reimerink, Michel Molier,
Jan H.M. Karregat, Johan IJlst, Věra M.J. Novotný, René A.W. van Lier, Hans L. Zaaijer

Supplementary Fig. 1 SARS-CoV-2 antibody signals (OD/CO ratios) using longitudinal samples, collected in April 2020 and prior to February 2020 (pre-outbreak). Signals are shown separately for seroconverting donors (left) and false-reactive (non-seroconverting) donors (right). To assess the consistency of false-reactive test results, archived samples of multiple associated pre-outbreak donations were tested, if available (right).

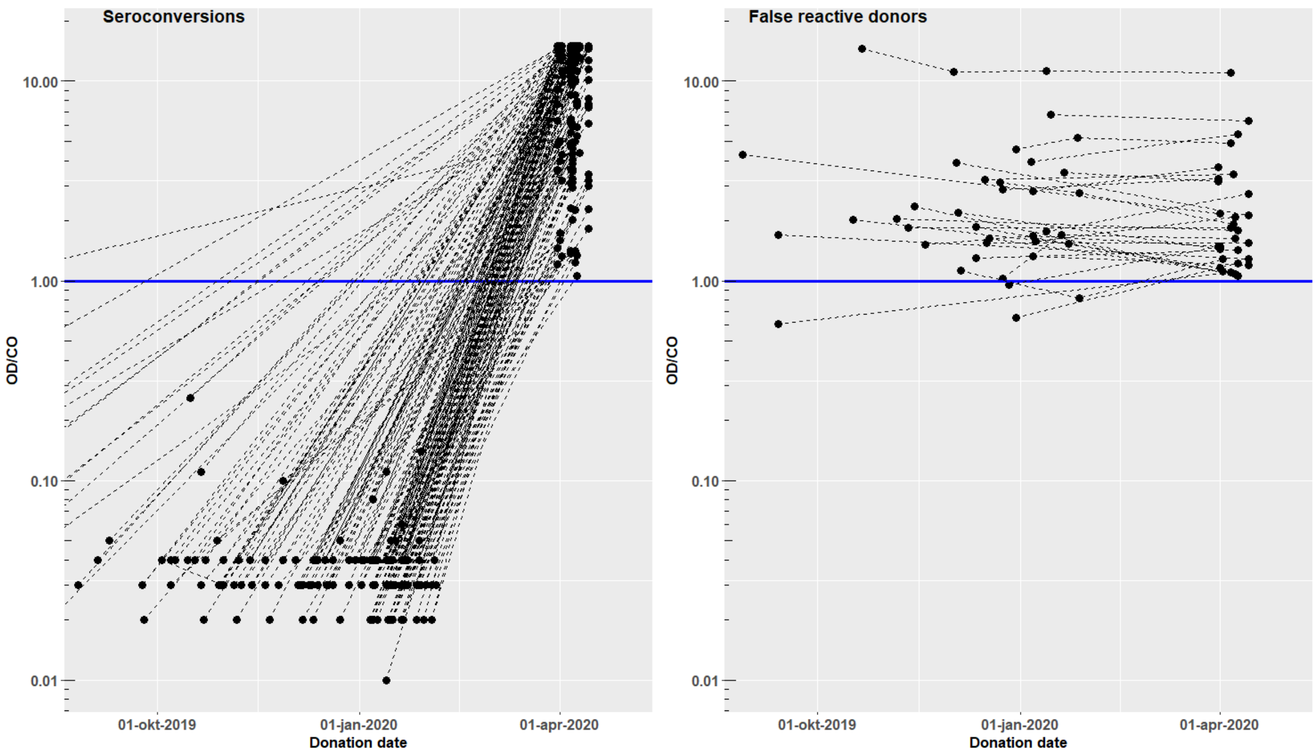

Supplementary Table 1 Prevalence of anti-SARS-CoV-2 antibodies in the Netherlands; 1-15 April 2020.
Numbers and percentages are given by region of residence (municipal health region), sex and age group.

Region of residence (municipal health region)	Donors tested	Positive	% Positive	False-reactive	Negative	Men	Women	Age 18-30 yrs	Age 31-40 yrs	Age 41-50 yrs	Age 51-60 yrs	Age 61-72 yrs	General population	Tested per 10,000
Brabant-Zuid Oost	105	10	9.5%	0	95	7/65 (10.8%)	3/40 (7.5%)	2/16 (12.5%)	0/12 (0.0%)	1/23 (4.3%)	5/18 (27.8%)	2/36 (5.6%)	766,745	1.37
Noord en Midden Limburg	277	19	6.9%	1	257	9/136 (6.7%)	10/141 (7.1%)	3/27 (11.1%)	3/25 (12.0%)	4/58 (6.9%)	6/96 (6.3%)	3/71 (4.2%)	518,155	5.35
Hart voor Brabant	617	36	5.8%	0	581	20/295 (6.8%)	16/322 (5.0%)	8/82 (9.8%)	3/78 (3.8%)	5/119 (4.2%)	8/184 (4.3%)	12/154 (7.8%)	1,058,365	5.83
West-Brabant	216	9	4.2%	0	207	3/116 (2.6%)	6/100 (6.0%)	2/22 (9.1%)	0/15 (0.0%)	1/53 (1.9%)	5/68 (7.4%)	1/58 (1.7%)	702,870	3.07
Zuid Limburg	445	16	3.6%	1	428	8/212 (3.8%)	8/233 (3.4%)	3/55 (5.5%)	1/56 (1.8%)	1/76 (1.3%)	4/136 (2.9%)	7/122 (5.7%)	599,000	7.43
Twente	254	9	3.5%	2	243	5/128 (3.9%)	4/126 (3.2%)	4/56 (7.1%)	2/41 (4.9%)	0/40 (0.0%)	1/65 (1.5%)	2/52 (3.8%)	627,745	4.05
Utrecht	514	18	3.5%	4	492	9/280 (3.2%)	9/234 (3.8%)	5/63 (7.9%)	3/68 (4.4%)	4/114 (3.5%)	4/155 (2.6%)	2/114 (1.8%)	947,960	5.42
Amsterdam	183	6	3.3%	3	174	2/79 (2.5%)	4/104 (3.8%)	3/56 (5.4%)	0/38 (0.0%)	2/32 (6.2%)	0/28 (0.0%)	1/29 (3.4%)	1,046,450	1.75
Gelderland-Zuid	276	9	3.3%	0	267	7/140 (5.0%)	2/136 (1.5%)	2/76 (2.6%)	0/27 (0.0%)	0/37 (0.0%)	3/67 (4.5%)	4/69 (5.8%)	553,630	4.99
Noord en Oost Gelderland	279	8	2.9%	0	271	4/132 (3.0%)	4/147 (2.7%)	1/38 (2.6%)	1/33 (3.0%)	3/58 (5.2%)	1/92 (1.1%)	2/58 (3.4%)	821,125	3.40
Volksgezondheid Utrecht	273	7	2.6%	0	266	4/135 (3.0%)	3/138 (2.2%)	4/120 (3.3%)	1/46 (2.2%)	1/37 (2.7%)	0/40 (0.0%)	1/30 (3.3%)	347,480	7.86
Hollands Noorden	340	8	2.4%	1	331	3/178 (1.7%)	5/162 (3.1%)	1/53 (1.9%)	2/22 (9.1%)	2/61 (3.3%)	1/112 (0.9%)	2/92 (2.2%)	655,200	5.19
Kennemerland	148	3	2.0%	2	143	2/65 (3.1%)	1/83 (1.2%)	0/24 (0.0%)	0/19 (0.0%)	1/39 (2.6%)	1/40 (2.5%)	1/26 (3.8%)	541,620	2.73
Zuid Holland-Zuid	323	6	1.9%	1	316	3/172 (1.7%)	3/151 (2.0%)	2/61 (3.3%)	1/50 (2.0%)	1/61 (1.6%)	1/89 (1.1%)	1/62 (1.6%)	490,180	6.59
Groningen	216	4	1.9%	2	210	2/104 (1.9%)	2/112 (1.8%)	0/76 (0.0%)	1/23 (4.3%)	0/25 (0.0%)	1/48 (2.1%)	2/44 (4.5%)	582,940	3.71
IJsselland	328	6	1.8%	2	320	2/167 (1.2%)	4/161 (2.5%)	3/43 (7.0%)	1/44 (2.3%)	1/65 (1.5%)	0/103 (0.0%)	1/73 (1.4%)	523,560	6.26
Zaanstreek/Waterland	221	4	1.8%	1	216	4/112 (3.6%)	0/109 (0.0%)	0/32 (0.0%)	1/27 (3.7%)	0/38 (0.0%)	2/78 (2.6%)	1/46 (2.2%)	334,740	6.60
Hollands Midden	657	10	1.5%	4	643	6/407 (1.5%)	4/250 (1.6%)	5/125 (4.0%)	1/77 (1.3%)	0/102 (0.0%)	3/196 (1.5%)	1/157 (0.6%)	794,340	8.27
Zeeland	238	3	1.3%	1	234	1/134 (0.7%)	2/104 (1.9%)	1/33 (3.0%)	0/25 (0.0%)	2/45 (4.4%)	0/69 (0.0%)	0/66 (0.0%)	382,325	6.23
Haaglanden	399	5	1.3%	2	392	3/228 (1.3%)	2/171 (1.2%)	3/61 (4.9%)	1/56 (1.8%)	0/70 (0.0%)	1/119 (0.8%)	0/93 (0.0%)	1,093,180	3.65
Drenthe	158	1	0.6%	0	157	0/74 (0.0%)	1/84 (1.2%)	0/17 (0.0%)	0/18 (0.0%)	0/32 (0.0%)	1/56 (1.8%)	0/35 (0.0%)	492,005	3.21
Gelderland Midden	203	1	0.5%	1	201	0/109 (0.0%)	1/94 (1.1%)	0/26 (0.0%)	0/22 (0.0%)	1/40 (2.5%)	0/59 (0.0%)	0/56 (0.0%)	685,150	2.96
Rotterdam-Rijnmond	237	1	0.4%	0	236	1/136 (0.7%)	0/101 (0.0%)	0/42 (0.0%)	0/27 (0.0%)	1/36 (2.8%)	0/74 (0.0%)	0/58 (0.0%)	1,303,205	1.82
Fryslân	389	1	0.3%	2	386	0/199 (0.0%)	1/190 (0.5%)	0/42 (0.0%)	0/27 (0.0%)	0/82 (0.0%)	0/116 (0.0%)	1/122 (0.8%)	647,260	6.01
Flevoland	34	0	0.0%	0	34	0/20 (0.0%)	0/14 (0.0%)	0/4 (0.0%)	0/4 (0.0%)	0/4 (0.0%)	0/13 (0.0%)	0/9 (0.0%)	411,675	0.83
Gooi en Vechtstreek	26	0	0.0%	0	26	0/17 (0.0%)	0/9 (0.0%)	0/1 (0.0%)	0/2 (0.0%)	0/5 (0.0%)	0/9 (0.0%)	0/9 (0.0%)	252,975	1.03
Abroad (Belgium, Germany)	5	0	0.0%	0	5	0/4 (0.0%)	0/1 (0.0%)	-	-	0/2 (0.0%)	0/2 (0.0%)	0/1 (0.0%)	n.a.	n.a.
Total	7,361	200	2.7%	30	7,131	105/3,844 (2.7%)	95/3,517 (2.7%)	52/1,251 (4.2%)	22/882 (2.5%)	31/1,354 (2.3%)	48/2,132 (2.3%)	47/1,742 (2.7%)	17,179,880	4.28
